# Disruption of *Trib1* Results in Granulosa Cells Steroid Hormone Synthesis Dysfunction and Infertility in Female Mice via Downregulations of *FOSL2* Expression

**DOI:** 10.3390/ani16081172

**Published:** 2026-04-11

**Authors:** Weibing Lv, Dan Zhao, Xinmiao Li, Gaga Shama, Hanzhuo Hu, Yilin Fan, Xianrong Xiong, Shi Yin, Jian Li, Yan Xiong

**Affiliations:** 1 College of Animal and Veterinary Sciences, Southwest Minzu University, Chengdu 610041, China; lwb18794850948@163.com (W.L.);; 2 Key Laboratory of Qinghai-Tibetan Plateau Animal Genetic Resource Reservation and Utilization of Ministry of Education, Southwest Minzu University, Chengdu 610041, China; 3 Key Laboratory of Qinghai-Tibetan Plateau Animal Genetic Resource Reservation and Utilization of Sichuan Province, Southwest Minzu University, Chengdu 610041, China; 4 Key Laboratory for Animal Science of National Ethnic Affairs Commission, Southwest Minzu University, Chengdu 610041, China

**Keywords:** *Trib1*, *FOSL2*, ovary, granulosa cells, steroidogenesis

## Abstract

Proper steroid hormone synthesis is essential for maintaining fertility in female animals. Tribbles pseudokinase 1 (*Trib1*), a member of the Tribbles pseudokinase family, exerts its functions mainly through interacting with other molecules. Numerous studies have shown that *Trib1* plays a central role in regulating cell proliferation. In mammals, the proliferation of granulosa cells (GCs) is a hallmark event in follicular development, which is essential for follicular maturation and successful ovulation. However, whether *Trib1* regulates ovarian steroid hormone synthesis remains largely unexplored. In this study, we found that *Trib1* is predominantly expressed in ovarian GCs. Knockdown of *Trib1* in GCs significantly reduced their capacity for steroid hormone synthesis. Furthermore, *Trib1* KO female mice were completely infertile, exhibiting impaired transition from primary to antral follicles, increased follicle atresia, and defective steroid hormone secretion. Ovarian RNA-Seq analysis revealed that differential expressed genes (DEGs) were significantly enriched in cholesterol metabolism and steroid biosynthesis pathways following *Trib1* deletion. Notably, *FOSL2*, a transcription factor that potentially bound to the promoters of the pivotal steroidogenic genes *Star* and *Cyp11a1*, was significantly down-regulated in *Trib1* KO mice. Crucially, overexpression of *FOSL2* in *Trib1*-deficient GCs restored *Star* and *Cyp11a1* expression and significantly rescued the ability of steroid hormone synthesis in GCs. Our findings unveil a novel *Trib1* gene governing steroidogenesis in GCs and is essential for fertility in female mice, providing profound insights into the female reproductive endocrinology and potential therapeutic targets.

## Simple Summary

Current research on *Trib1* biology has largely focused on human metabolic diseases, with *Trib1* notably linked to cell proliferation. However, the role of *Trib1* in ovarian granulosa cell (GC) function and folliculogenesis remains unclear. To investigate this, we generated *Trib1* knockout mice and found that *Trib1* deficiency leads to female infertility, characterized by impaired folliculogenesis and defective steroid hormone secretion. Our findings reveal a novel role for *Trib1* in governing steroidogenesis in granulosa cells and demonstrate that *Trib1* is essential for fertility in female mice, providing important insights into female reproductive endocrinology and highlighting potential therapeutic targets.

## 1. Introduction

The ovaries function as the reproductive gonads in female mammals and are integral to multiple aspects of female development and physiology [1]. The follicle is recognized as both a structural and functional unit of the mammalian ovary, comprising oocytes and granulosa cells (GCs) [2]. GCs encase the oocyte and play a crucial role in providing essential nutrients and hormones, which are vital for oocyte growth, development, and maturation [3]. In recent years, significant progress has been made in understanding the regulatory mechanisms underlying oogenesis, follicular genesis, ovarian atresia, and steroidogenesis, particularly through the studies of ovarian physiological function. GCs have been shown to play a central role in follicular growth, development, and follicular atresia by regulating processes such as proliferation, differentiation, the cell cycle, and apoptosis [4].

The Tribbles family is classified as a pseudoprotein kinase family, which belongs to the human Ca^2+^/calmodulin-dependent protein kinase (CAMK) subfamily [5]. This family comprises three homologous members: Tribbles homolog 1 (*Trib1*), Tribbles homolog 2 (*Trib2*), and Tribbles homolog 3 (*Trib3*). All members of the Tribbles family possess a distinctive kinase-like domain, which is flanked by a variable N-terminus and a predominantly conserved C-terminal COP1 binding site [6]. Numerous studies have indicated that *Trib1* functions as a scaffold protein, facilitates protein–protein interactions and thereby promotes post-translational modification such as phosphorylation or ubiquitination of target proteins [7,8]. Furthermore, *Trib1* has been shown to inhibit cell growth, proliferation, and differentiation during normal developmental processes [9]. Recent research on the Tribbles family has increasingly focused on its roles in tumorigenesis, inflammation [10] and lipid metabolism [11]. Notably, emerging evidence indicates that TRIB family members play critical roles in female reproduction [12]. For instance, *Trib1* participates in progesterone synthesis in yak ovarian GCs, although its precise molecular mechanism remains unclear [13]. Additionally, *Trib2* is differentially expressed in GCs of follicles at various developmental stages and may regulate follicular development and ovarian function [14].

Previous studies by our team demonstrated that the TRIB1 protein is significantly more highly expressed in ovarian GCs than in other cell types in the yak ovary, suggesting its potential crucial role in GCs [13]. However, the precise function and underlying molecular mechanisms by which *Trib1* regulates steroidogenesis in ovary GCs remain unclear. To further investigate the role of the *Trib1* gene in regulating steroid hormone secretion in ovarian GCs and its effects on reproductive performance, this study generated a global *Trib1* knockout (*Trib1* KO) mouse model (C57BL/6J background) using CRISPR/Cas9 technology. Follicle counts at different developmental stages, breeding performance, ovarian RNA sequencing (RNA-Seq), and rescued experiments were conducted to comprehensively elucidate the role and potential mechanisms of *Trib1* in regulating mammalian steroidogenesis, follicular development, and litter size. This study provides a theoretical foundation for targeting the *Trib1* gene to improve fertility in female mammals.

## 2. Materials and Methods

### 2.1. Construction, Genotyping, Husbandry, and Breeding Data Analysis of Trib1 Knockout Mice

*Trib1* KO mice were generated by Sai Ye Biotechnology Co., LTD (Suzhou, China). The *Trib1* KO mice were crossed with wild-type C57BL/6 mice to produce heterozygous offspring. Genotyping of *Trib1* KO mice was performed by PCR on tail genomic DNA, with primer sequences listed in Table 1. Subsequently, the mice were intercrossed to generate *Trib1* KO homozygous mice (*Trib1*−/−, KO), heterozygous mice (*Trib1*+/−, HE), and wild-type mice (*Trib1*+/+, WT). All experimental mice were maintained in the SPF animal facility of Southwest Minzu University at about 22–26 °C under a 12 h light/dark cycle, with ad libitum access to standard chow and water.

For fertility assessment, *Trib1*−/−, *Trib1*+/−, female and *Trib1*+/+ male mice aged 8 to 10 W were mated with each other and were kept for 6 months (6M) each. The number of offspring, genotype, and weight of each pregnant female were recorded after birth.

### 2.2. Collection of Mouse Ovaries, Tissue, and Serum Samples

Genotype, age, and sex of the mice were verified, and body weight was recorded. Blood samples were collected via retro-orbital bleeding, allowed to clot for 40 min, centrifuged to obtain serum, aliquoted, and stored at −80 °C. Following dissection, tissues including the heart, liver, spleen, lungs, kidneys, and fat were collected. Ovarian samples were also collected, and their weight was recorded. Samples designated for total RNA extraction were stored at −80 °C, whereas ovarian tissues for histological analysis were fixed in 4% paraformaldehyde (PFA) and stored at 4 °C.

### 2.3. Hematoxylin and Eosin (H&E) Staining of Ovarian Sections

To assess the impact of *Trib1* KO on follicular development in mouse ovaries, we conducted estrous cycle identification on 9-week-old (9 W) female *Trib1*−/− mice and their *Trib1*+/+ counterparts. During the follicular phase (proestrus–estrus with rising estrogen) of the estrous cycle, we dissected the mice and collected their ovarian tissues, which were immediately fixed in 4% paraformaldehyde (PFA) for 24 h. The ovarian tissue was embedded and stained with H&E staining. The images were taken with an inverted microscope.

### 2.4. Total RNA Extraction and Reverse Transcription Quantitative Polymerase Chain Reaction (RT-qPCR)

Total RNA was extraction from tissues and cells using Trizol (Vazyme, Nanjing, China) according to the manufacturer’s instructions and subsequently reverse transcribed with HiScript II Q RT SuperMix for qPCR (+gDNA wiper) (Vazyme, Nanjing, China). The CFX96™ system (Bio-Rad, Hercules, California, USA) was used with 2 X SYBR Green qPCR Mixture (HLINGENE, Shanghai, China) with the following program steps: 94 °C for 2 min; 45 cycles of 94 °C for 15 s, 60 °C for 15 s, and 72 °C for 30 s; followed by dissociation curve and cool down. Each sample analysis was repeated independently in triplicate. Using *GAPDH* as the internal reference gene, relative gene mRNA expression was calculated with the 2^−ΔΔCt^ method. The primer information for the genes is provided in Table 2 as follows:

### 2.5. Quantification of Ovarian Follicle Counts at Different Developmental Stages in Mice

Ovaries fixed in 4% paraformaldehyde for 24 h were washed, dehydrated through a graded ethanol series (70%, 85%, 95%, 100%), cleared in xylene, and embedded in paraffin in metal molds. After embedding and cooling, serial sections of 5–8 μm were cut from the ovarian apex. The first section containing a clearly identifiable secondary follicle was designated Section 1; thereafter, every 10th section was labeled as the next section, continuing until the entire ovary was sectioned. Sections were deparaffinized with xylene and stained with H&E staining, and images were captured for morphological observation. Follicles at different developmental stages were counted by comparing successive numbered sections to avoid double counting. Only follicles with visible oocytes were counted to avoid duplication. Follicle classification was performed based on previously described criteria [15]. Each group included 6 biological replicates.

### 2.6. Immunohistochemistry

To study TRIB1 protein expression in ovarian tissue, wild-type mouse ovary sections were subjected to immunohistochemistry. Antigen retrieval was performed by heating sections in 0.01 M citrate buffer to boil for 15 min. After retrieval, sections were washed in PBS (pH 7.4) for 5 min, three times. Endogenous peroxidase activity was blocked with 3% BSA for 30 min at room temperature. The TRIB1 primary antibody (TRB-1 (E-7): sc-393536, Santa Cruz, California, USA) was diluted 1:100 and applied to the sections to cover the tissue, followed by incubation in a humidified chamber at 4 °C for 12 h. Sections were washed with PBS three times, then incubated with secondary antibody (goat anti-mouse, GB23301, Servicebio, Wuhan, China) diluted 1:3000 for 1 h at room temperature, and color developed with freshly prepared DAB. The slides were rinsed with water to stop the reaction, counterstained with hematoxylin, and washed until clear. Dehydration was performed through graded ethanol (70%, 80%, 90%, 100%), and sections were mounted with neutral resin. Images were captured using a Zeiss LSM800 confocal microscope, and immunohistochemistry density was analyzed with Image-Pro Plus 6.0.

### 2.7. Isolation and Culture of GCs from Mouse Ovaries

Female mice (3–4 weeks old) were intraperitoneally injected with 10 IU PMSG (Ningbo Second Hormone Factory, Ningbo, Zhejiang, China). Forty-eight hours after the injection, ovaries were collected and placed in 50 μL of cell complete medium (DEME/Ham’s F12 supplemented with 1% penicillin and streptomycin and 10% fetal bovine serum). Under the field of view of the anatomical microscope, the sinus follicles were punctured with a fine needle to release follicular GCs. After collecting the follicular fluid containing granulosa cells into centrifuge tubes, we centrifuged it at 800× *g* for 5 min to obtain the granulosa cell pellet. The cells were then resuspended using the commercially available Mouse Ovarian Granulosa Cell Complete Medium (catalog number CM-M050, Wuhan Pricella Biotechnology Co., Ltd., Wuhan, China) and seeded into culture dishes. The dishes were gently shaken back and forth several times on the workbench using the “cross method” (moving slowly from left to right and top to bottom, following the steps of drawing a cross). Immediately afterward, the dishes were placed into an incubator set at 37 °C with 5% CO_2_. The cell status was observed at 24 h and 48 h intervals.

### 2.8. Cell Immunofluorescence Staining

Cells at 80% confluence were fixed with 4% PFA for 20 min and permeabilized with 100 mM glycine solution for 10 min. Blocking was performed for 45–60 min. Primary antibodies were incubated overnight at 4 °C. DAPI and species-specific fluorescent secondary antibodies were added and incubated at 37 °C for 45 min. Images were captured under a fluorescence microscope. For mouse immunofluorescence staining, primary antibodies were diluted as follows: TRIB1 (1:100), and FSHR (1:200). Fluorescent secondary antibodies were diluted 1:2000.

### 2.9. Cell Transfection Experiment

siRNA targeting the mouse *Trib1* CDS (coding sequence) and a negative control (NC) sequence were designed, with detailed sequences provided in Table 3. An overexpression plasmid for mouse *FOSL2* was constructed using the pcDNA3.1 vector. Both siRNA and the overexpression plasmid were synthesized by Gene Pharma (Shanghai, China). GCs were seeded in 24- or 6-well plates and transfected at 70–80% confluence following the manufacturer’s protocol for the Lipofectamine™ 3000 kit (Invitrogen, L3000-015, Carlsbad, CA, USA). Culture media and cells were collected 48 h after transfection for subsequent experiments.

### 2.10. Enzyme-Linked Immunosorbent Assay (ELISA)

The concentrations of estradiol (E2) and progesterone (P4) in both mouse serum and the supernatant of the mouse ovarian GCs culture medium were detected with the ELISA kit (Jiangsu Meimian Industrial Co., Ltd, Yancheng, Jiangsu, China) according to the instructions. The standard curve was fitted with R2 of 0.99, and then the content of E2 and P4 was calculated according to the OD value obtained by detection according to the standard curve. The sensitivity for P4 and E2 determinations was 0.1 nmol/mL and 0.1 pmol/L, respectively. The intra-assay coefficient of variation was <10%, and the inter-assay coefficient of variation was <10%.

### 2.11. RNA-Seq Differential Gene Expression (DEGs) Analysis and Pathway Analysis

*Trib1*−/− and *Trib1*+/+ mice were generated by breeding. Estrous-cycle staging identified those in the follicular phase. Ovarian samples were collected from *Trib1*−/− and *Trib1*+/+ mice (*n* = 3; 2 M old; littermates were paired for comparison) and total RNA was extracted from all samples using TRIzol. The integrity of the RNA was verified using the Agilent 2100 Bioanalyzer system (2100, Agilent, CA, USA). Each sample (1.5 μg) was sequenced using the Illumina platform. After quality control of the raw data, HISAT2 (Version 2.1.0) was used for alignment with the reference genome. The number of reads mapped to protein-coding genes in each sample was obtained using HTSeq-count software (Version 0.11.2), and FPKM (Fragments Per Kilobase of transcript per Million mapped reads) values were calculated to determine gene expression levels based on the FPKM formula. Differential gene expression analysis was performed using DESeq2 (Version 1.22.2) with default conditions (*p* Value < 0.05 and fold change > 2 or fold change < 0.5) for gene selection. Gene Ontology (GO) enrichment analysis of differentially expressed genes (DEGs) was conducted using ClusterProfiler (Version 3.8.1), and statistical enrichment in Kyoto Encyclopedia of Genes and Genomes (KEGG) pathways was assessed. Twelve randomly selected differential genes from the differential gene set were validated using RT-qPCR, with primer sequences provided in Table 4.

### 2.12. Statistical Analysis

The results were graphed using GraphPad Prism 8 software, and SPSS 25.0 was used for the single-factor analysis of variance for multiple comparisons and significance analysis. All data were expressed as mean ± standard error of mean (Mean ± SEM).

## 3. Results

### 3.1. Expression Pattern and Localization of Trib1 in the Ovary

**Figure 1:**
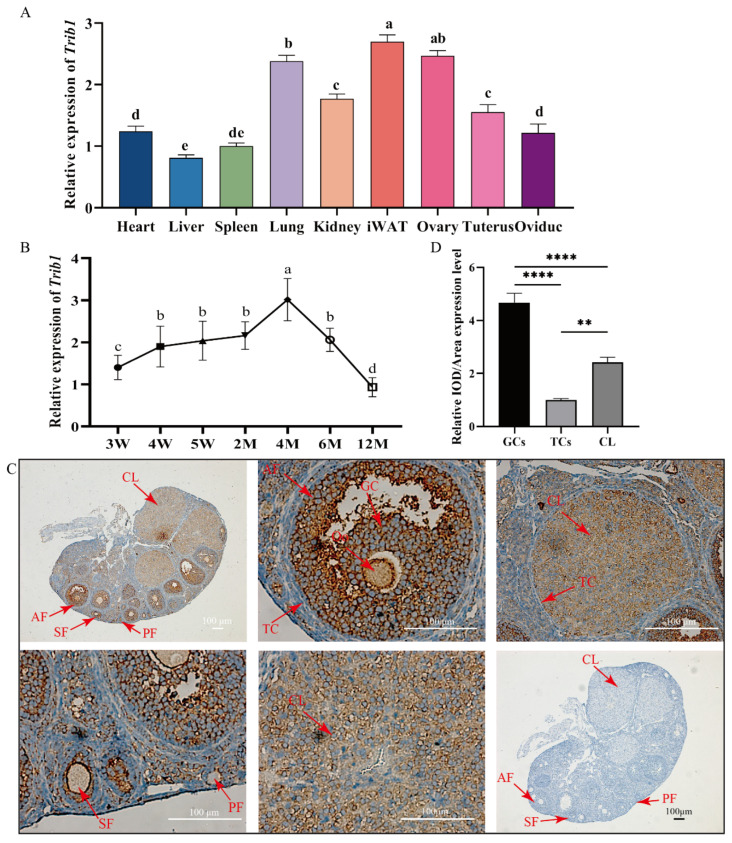
Expression Pattern of *Trib1* in the Ovary and Localization of TRIB1 Protein in Ovarian Tissue. (**A**) Expression levels of *Trib1* across various tissues in follicular phase mice (C57BL/6J background) (*n* = 6). (**B**) Expression of *Trib1* in the ovaries of female mice at different developmental stages during the follicular phase (*n* = 6). W: Weeks; M: Months. (**C**) Localization and expression of TRIB1 protein in metestrus mouse ovaries and its negative control in immunohistochemical staining. The primary antibody for TRIB1 was diluted at 1:100. Oo: Oocyte; GC: Granulosa cells; TC: Theca cells; CL: Corpus luteum. PF: primary follicles; SF: secondary follicles; AF: antral follicles. Scale bar: 100 μm. (**D**) Relative mean optical density (IOD/area) of TRIB1 protein in GCs, theca cells, and corpus luteum (*n* = 6). Different lowercase letters indicate significant differences (*p* < 0.05), while the same letters indicate no significant difference (*p* > 0.05). ** *p*  <  0.01, **** *p* < 0.0001.

To investigate the expression pattern of *Trib1* across various mouse tissues, its relative expression levels were compared. The results show that *Trib1* was highly expressed in inguinal white adipose tissue (iWAT) and the ovary (Figure 1A). Among the reproductive organs of female mice, *Trib1* expression in the ovary was significantly higher than in other tissues (*p* < 0.05) (Figure 1A). To further investigate the age-related expression of *Trib1* in the ovaries of female mice, ovarian tissues were collected from 3-week-old (3W), 4W, 5W, 2M, 4M, 6M, and 12M female mice during the follicular phase. Compared to the 3W group, *Trib1* expression was significantly elevated in the ovaries at 4W, 5W, and 2M (*p* < 0.05), reaching the highest level at 4M, which was significantly higher than that of other stages (*p* < 0.05). With increasing age, *Trib1* expression significantly declined at 6M and reached the lowest level at 12M (*p* < 0.05) (Figure 1B). To study the localization and expression of TRIB1 protein in adult mouse ovarian tissue, we selected ovarian sections from 2M mice in metestrus and performed immunohistochemical staining. The results show that TRIB1 protein was predominantly expressed in follicles at different developmental stages, with limited expression in ovarian stromal cells, compared to the negative control group (Figure 1C). Further quantitative analysis revealed that, in follicles at different developmental stages, TRIB1 protein expression in GCs was significantly higher than in theca cells (TCs) and corpus luteum (CL) (*p* < 0.0001) (Figure 1D). Additionally, TRIB1 expression in CL was significantly higher than in TCs (*p* < 0.01) (Figure 1D). These findings indicate that the *Trib1* gene might play a key role in GCs, contributing to follicular development and the maintenance of normal ovarian function.

### 3.2. Knockdown of Trib1 in GCs Inhibits Steroid Hormone Biosynthesis

**Figure 2:**
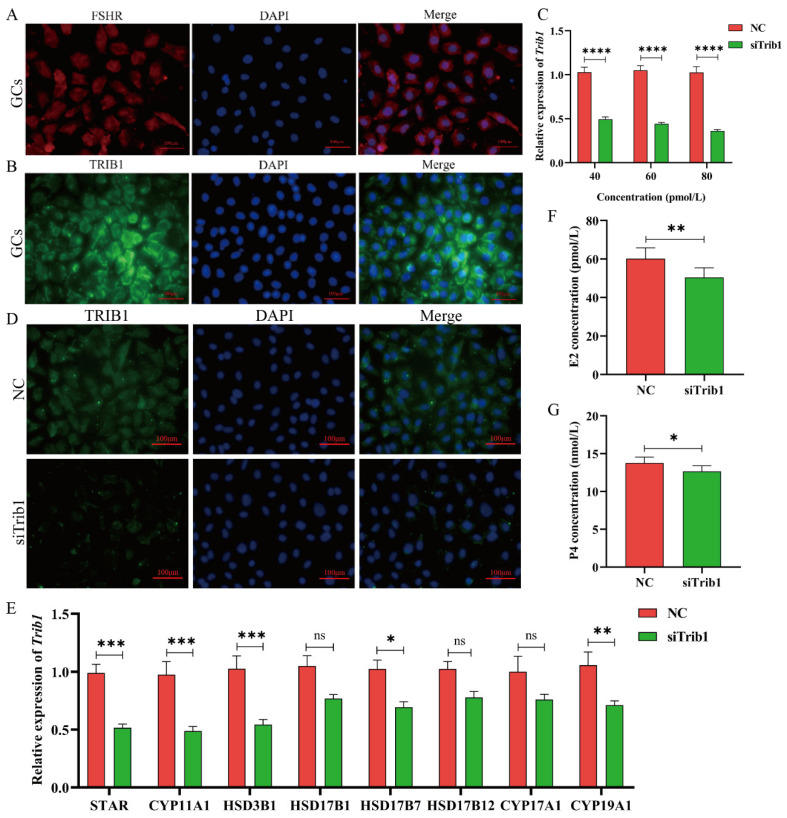
Reduced *Trib1* Expression in GCs Inhibits Steroid Hormone Biosynthesis. (**A**) Identification of mouse ovarian GCs using FSHR antibody. The primary antibody for FSHR was diluted at 1:200. Scale bar: 100 μm. (**B**) Localization of TRIB1 protein in ovarian GCs. The primary antibody for TRIB1 was diluted at 1:100. Scale bar: 100 μm. (**C**) The knockdown efficiency of the *Trib1* gene was measured in GCs at 48 h after transfection with different siRNA concentrations (*n* = 6). (**D**) Immunofluorescence staining of TRIB1 in GCs 48 h after siRNA transfection. The primary antibody for TRIB1 was diluted at 1:100. Scale bar: 100 μm. (**E**) Expression levels of steroidogenesis-related genes in GCs 48 h after siRNA transfection. (**F**,**G**) Estradiol (**F**) and Progesterone (**G**) concentrations in the cell culture medium from the NC and siTrib1 groups (*n* = 6). * *p* < 0.05, ** *p* < 0.01, *** *p* < 0.001, **** *p* < 0.0001, ns: *p* > 0.05.

The primary function of ovarian GCs is the secretion of steroid hormones, which are critical for follicular development. To further investigate the function of *Trib1* in GCs, mouse GCs were isolated and cultured in vitro and identified using an FSH receptor (FSHR) antibody. Immunofluorescence analysis showed that over 96% of the cultured cells were FSHR-positive, indicating suitability for subsequent experiments (Figure 2A). TRIB1 protein immunofluorescence staining demonstrated that the protein was localized in both the nucleus and cytoplasm of GCs, with predominant cytoplasmic localization (Figure 2B). To knock down *Trib1* expression in GCs, siRNA (siTrib1) was transfected into GCs and 80 pM of siTrib1 reduced *Trib1* expression by over 60% (Figure 2C). Consistently, siTrib1 treatment significantly inhibited Trib1 protein expression as seen with immunofluorescence staining analysis (Figure 2D). *Trib1* knockdown significant downregulation key steroidogenesis-related genes, including *Star*, *Cyp11a1*, *Hsd3b1*, *Hsd17b7*, and *Cyp19a1* (Figure 2E). Next, we assessed the impact of *Trib1* knockdown on steroid hormone secretion. The results show that *Trib1* knockdown significantly reduced estradiol (E2) secretion (*p* < 0.01) (Figure 2F) and also decreased progesterone (P4) secretion (*p* < 0.05) (Figure 2G). These findings suggest that *Trib1* knockdown inhibits steroidogenic genes expression, impairing steroid hormones synthesis and reducing E2 and P4 secretion.

### 3.3. Deletion of Trib1 Results in Impaired Reproductive Performance in Female Mice

**Figure 3:**
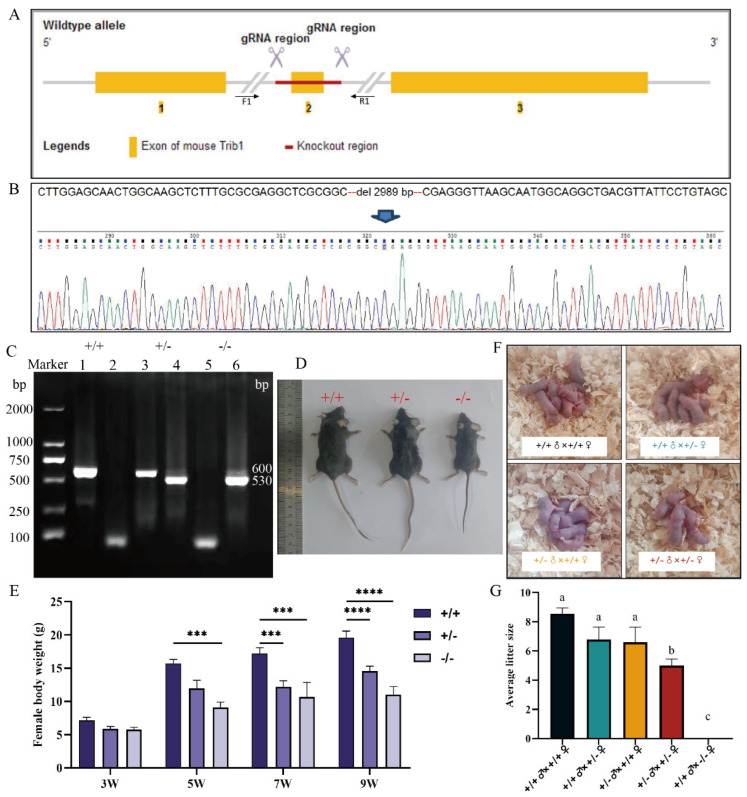
Trib1 gene knockout results in delayed development and severely impaired reproductive performance in female mice. (**A**) Schematic diagram of the construction of *Trib1* KO mice. (**B**) DNA sequencing results of *Trib1* KO mice. (**C**) Genotyping of mice. Lanes 1, 3, and 5 show PCR products amplified with the F1/R1 primers, and lanes 2, 4, and 6 show products amplified with the F1/R2 primers. (**D**) Photographs of 9 W female mice of different genotypes. (**E**) Body weight statistics of mice from 3 W to 9 W (*n* = 12). (**F**) Photographs of offspring obtained from breeding mice of different genotypes. (**G**) Average litter size from breeding mice of different genotypes (*n* = 12, recorded over 6 M). *** *p* < 0.001, **** *p* < 0.0001. Different lowercase letters indicate significant differences (*p* < 0.05), while the same letters indicate no significant difference (*p* > 0.05).

To investigate the biological function of *Trib1* in the mouse ovary, we employed CRISPR/Cas9 gene-editing technology to delete the second exon of the *Trib1* gene (Figure 3A). Following the generation *Trib1* heterozygous knockout (*Trib1*+/−) mice, offspring were produced through breeding and subsequently genotyped (Figure 3C). Based on genotyping results, mice were classified as wild-type (*Trib1*+/+), heterozygous (*Trib1*+/−), or homozygous knockout (*Trib1*−/−) (Figure 3C). DNA sequencing of *Trib1*−/− mice confirmed the deletion of a 2989 bp fragment (Figure 3B), confirming the successful generation of the *Trib1* KO model for subsequent analyses. Body weight analysis revealed no significant differences among genotypes at 3W (Figure 3E). However, *Trib1*+/− and *Trib1*−/− mice exhibited significantly lower body weights compared to *Trib1*+/+ mice by 7W and 9W (Figure 3D,E). Female and male mice of different genotypes (9W) were paired for continuous breeding over 6 M. The results show that *Trib1*+/− females bred with *Trib1*+/− males produced significantly fewer offspring compared to wild-type pairings (*p* < 0.05), whereas *Trib1*−/− females were completely infertile (Figure 3F,G). Analysis of the genotypes of offspring from *Trib1*+/− breeding pairs revealed a significant deviation from expected Mendelian ratios (*p* < 0.05), with the number of *Trib1*−/− offspring significantly lower than expected (Table 5). In summary, *Trib1* KO resulted in reduced growth, lower body weight, and complete infertility in female mice. These findings indicate that *Trib1* plays a critical role in regulating growth, development, and reproductive performance in mice.

### 3.4. Trib1 Gene Knockout Disrupts the Follicular Development Process and Reduces Steroid Hormone Secretion Capacity in Female Mice

**Figure 4:**
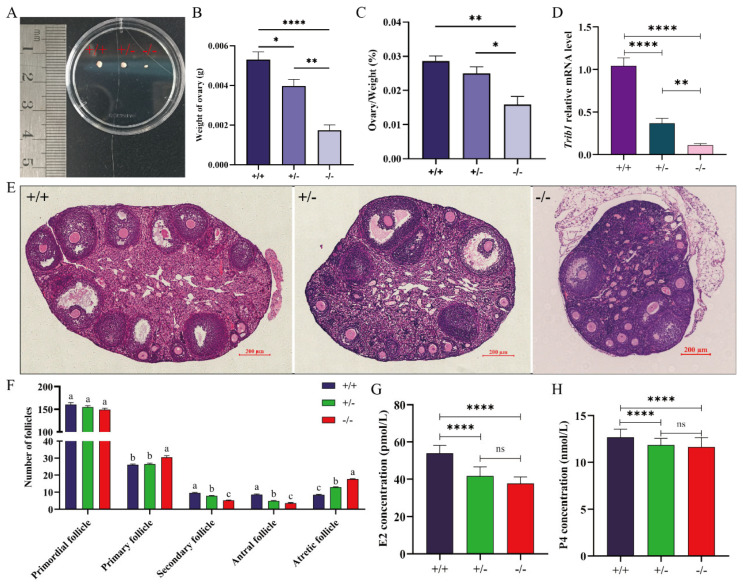
*Trib1* gene knockout disrupts the follicular development process in female mice, with GCs showing markedly reduces steroid hormone secretion capacity and significant decreases in ovarian weight and volume. (**A**) Representative images of ovaries from 9 W female mice in the follicular phase across different genotypes. (**B**) Statistical analysis of ovarian weight in 9 W female mice in the follicular phase across different genotypes (*n* = 6). (**C**) Statistical analysis of the ovarian weight-to-body weight ratio in 9 W female mice in the follicular phase across different genotypes (*n* = 6). (**D**) Relative *Trib1* mRNA expression levels in GCs of *Trib1* KO mice during the follicular phase detected by RT-qPCR (*n* = 6). (**E**) H&E staining of the largest cross-sections of ovaries from 9 W female mice in the follicular phase across different genotypes. Scale bar: 200 μm. (**F**) Statistical analysis of follicle numbers at different developmental stages in ovaries from 9 W female mice in the follicular phase across different genotypes (*n* = 6). Different lowercase letters indicate significant differences (*p* < 0.05), while the same letters indicate no significant difference (*p* > 0.05). (**G**) Serum E2 levels in 9 W female mice in the follicular phase across different genotypes (*n* = 6). (**H**) Serum P4 levels in 9 W female mice in the follicular phase across different genotypes (*n* = 6). * *p* < 0.05, ** *p* < 0.01, **** *p* < 0.0001, ns: *p* > 0.05.

The ovary is a critical organ in the mouse reproductive system, playing an essential role in reproduction and fertility. Dissection of 9 W female mice of different genotypes revealed that the ovaries of *Trib1*−/− mice were significantly smaller than those of other genotypes (Figure 4A). Further analysis showed that ovarian weight in *Trib1*−/− mice was markedly lower than in *Trib1*+/+ and *Trib1*+/− mice (*p* < 0.01) (Figure 4B), and the ovarian index (ovarian weight-to-body ratio) was also significantly reduced (*p* < 0.05) (Figure 4C). In vitro culture of ovarian GCs from mice of different genotypes showed that the relative expression of *Trib1* was significantly reduced in *Trib1*+/− and *Trib1*−/− mice compared to *Trib1*+/+ (Figure 4D), confirming the successful establishment of the *Trib1* KO model. H&E staining of ovaries revealed differences in maximum cross-sectional area, follicle number, and morphology among genotypes (Figure 4E). Although the number of primordial follicles showed no significant difference across genotypes, *Trib1*−/− ovaries exhibited a significantly higher number of primary follicles compared to *Trib1*+/+ and *Trib1*+/− ovaries (*p* < 0.05). In contrast, *Trib1*−/− ovaries exhibited significantly fewer secondary and mature follicles (*p* < 0.05), accompanied by a marked increase in atretic follicles (*p* < 0.05) (Figure 4F). These results indicate that disruption of *Trib1* blocks the transition from primary follicles to antral follicles and substantially increases follicular atresia, ultimately leading to ovarian dysfunction. Serum steroid hormone measurements further showed that E2 and P4 levels were significantly reduced in *Trib1*−/− and *Trib1*+/− mice compared to *Trib1*+/+ mice (*p* < 0.001) (Figure 4G,H). Together, these findings indicate that *Trib1* KO severely impairs ovarian function and disrupts follicular development, accompanied by a reduction in steroid hormone secretion.

### 3.5. Trib1 Deletion in Mouse Ovaries Affects the Gene Profiles Involved in Ovarian Steroid Hormone Biosynthesis Pathways

**Figure 5:**
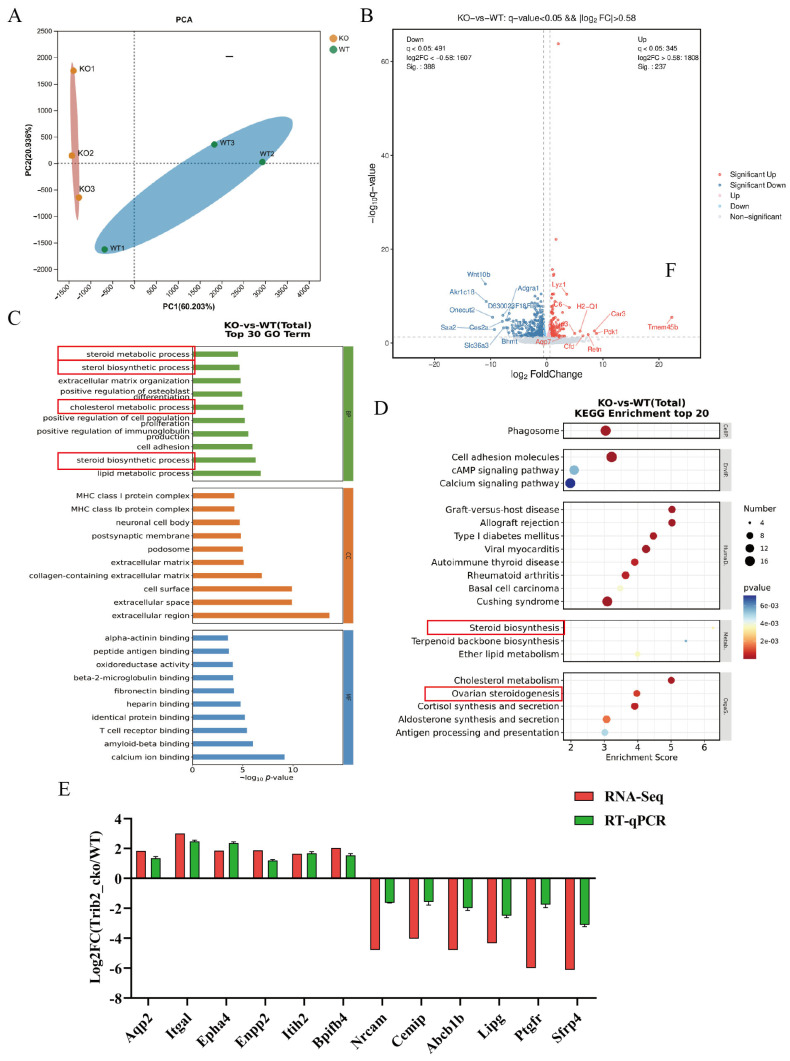
*Trib1* knockout impairs estrogen secretion and steroidogenesis gene expression. (**A**) PCA plot of ovarian RNA-Seq data. (**B**) Volcano plot of DEGs. (**C**) KEGG analysis of the top 20 enriched pathways of DEGs in mouse ovaries between WT and KO after RNA-Seq. (**D**) GO enrichment analysis (Top 30) of DEGs. (**E**) KEGG enrichment analysis (Top 20) of DEGs. (**F**) Validation of DEGs identified by RNA-Seq using RT-qPCR. All ovarian tissue samples used for RNA-Seq were collected during the follicular phase of the estrous cycle.

To investigate the potential molecular mechanisms of the *Trib1* gene in ovarian steroid hormone biosynthesis and ovarian function, ovarian tissues were collected from 9 W *Trib1*+/+ (WT) and *Trib1*−/− (KO) mice for RNA-Seq analysis. PCA results showed good reproducibility among samples within each group, with inter-group variance exceeding intra-group variance (Figure 5A). Using a threshold of *q*-value < 0.05 and |log2FC| > 0.58, 625 DEGs were identified, including 237 upregulated and 388 downregulated genes in the KO group compared to the WT group (Figure 5B). GO enrichment analysis of the DEGs revealed significant enrichment in pathways such as steroid metabolic process (GO:0008202, 14 genes), sterol biosynthetic process (GO:0016126, 7 genes), cholesterol metabolic process (GO:0008203, 14 genes), and steroid biosynthetic process (GO:0006694, 12 genes) (Figure 5C). KEGG pathway enrichment analysis further demonstrated that DEGs were mainly enriched in pathways such as steroid biosynthesis (mmu00100, 4 genes), ovarian steroidogenesis (mmu04913, 8 genes), and cholesterol metabolism (mmu04979, 8 genes) (Figure 5D). To validate the RNA-Seq results, 12 DEGs were randomly selected for RT-qPCR verification, which showed consistent expression patterns consistent with the RNA-Seq data, confirming their high reliability (Figure 5E). These findings indicate that *Trib1* knockout significantly affects mRNA transcriptional regulation in the ovary and disrupts the steroid hormone biosynthesis process, resulting in a marked reduction in ovarian E2 and P4 levels.

### 3.6. Trib1 Regulates Steroid Hormone Biosynthesis Through Modulating the Expression of FOSL2

**Figure 6:**
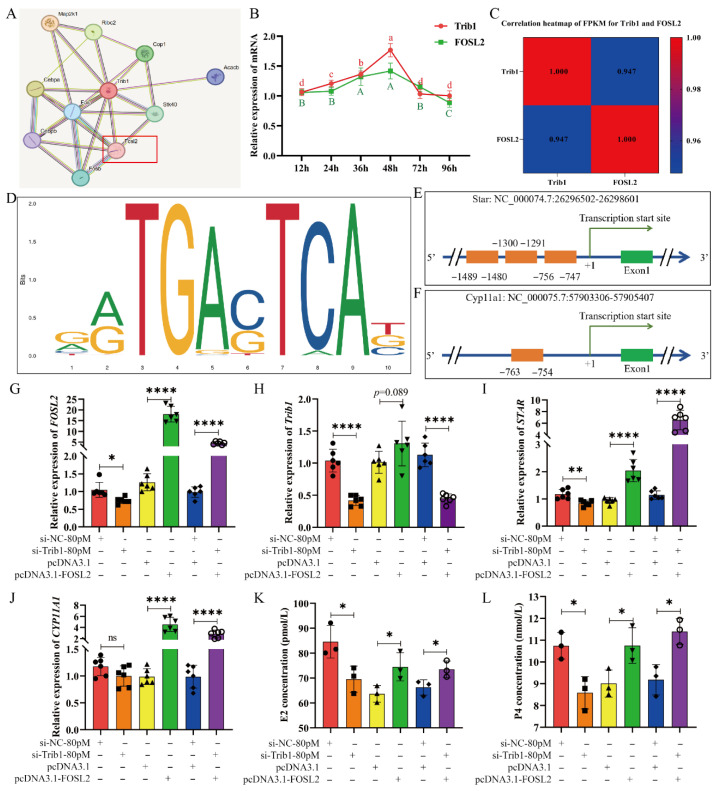
*Trib1* modulates FOSL2 expression to regulate steroid hormone biosynthesis. (**A**) Prediction of *Trib1*-interacting molecules using the STRING database. (**B**) Expression patterns of *Trib1* and *FOSL2* in cultured mouse ovarian GCs. Different lowercase letters indicate significant differences (*p* < 0.05), while the same letters indicate no significant difference (*p* > 0.05). (**C**) Correlation analysis of *Trib1* and *FOSL2* expression levels in mouse ovary RNA-Seq data. (**D**) Motif sequence of the *FOSL2* transcription factor. (**E**,**F**) Predicted binding site of the *FOSL2* motif in the promoter region of the *Star* (**E**) and *Cyp11a1* (**F**) gene. (**G**–**J**) Relative expression of *FOSL2* (**G**), *Trib1* (**H**), *Star* (**I**), and *Cyp11a1* (**J**) in mouse ovarian GCs after co-treatment with siTrib1 and *FOSL2* overexpression plasmid. (**K**,**L**) E2 (**K**) and P4 (**L**) concentration in mouse ovarian GCs after co-treatment with siTrib1 and *FOSL2* overexpression plasmid. * *p* < 0.05, ** *p* < 0.01, **** *p* < 0.0001, ns: *p* > 0.05.

*Trib1* is a member of the pseudokinase family and functions primarily through interacting with other molecules. To investigate proteins interacting with *Trib1* in ovarian GCs, we performed an interaction analysis using the STRING database, which identified the transcription factor *FOSL2* as an interacting partner of *Trib1* (Figure 6A). In cultured mouse ovarian GCs, *Trib1* and *FOSL2* expression patterns were highly consistent, both exhibiting increased expression with prolonged culture, peaking at 48 h and subsequently declining to the lowest level at 96 h (Figure 6B). Moreover, RNA-Seq data from mouse ovaries showed that *FOSL2* expression was significantly downregulated in the absence of *Trib1* (*p* < 0.05), with a high correlation coefficient of 0.947 between *Trib1* and *FOSL2* (Figure 6C), suggesting a close regulatory relationship in GCs.

To investigate whether *Trib1* affecting steroid hormone secretion in ovarian GCs through the *FOSL2* gene, we predicted the *FOSL2* motif sequence (Figure 6D) at the promoter regions (defined as −1899 bp to +100 bp relative to the transcription start site) of steroid hormone synthesis-related genes, including *Star*, *Cyp11a1*, *Hsd3b1*, *Hsd3b1*, *Hsd17b1*, *Hsd17b7*, *Hsd17b12*, *Cyp17a1*, and *Cyp19a1*. Binding sites with a Relative Score > 0.90 were identified at the promoter regions of *Star* (Figure 6E) and *Cyp11a1* (Figure 6F). Consistently, knockdown of *Trib1* significantly downregulated the mRNA levels of *Star* and *Cyp11a1* (Figure 2E). To validate the interaction between *Trib1* and *FOSL2* in the steroid hormone biosynthesis process of GCs, the rescued experiments were performed by co-transfecting siTrib1 and *FOSL2* overexpression plasmid into mouse ovarian GCs. Knockdown of *Trib1* in GCs significantly reduced *Trib1* expression (*p* < 0.0001), downregulated *FOSL2* (*p* < 0.05), and decreased *Star* expression (*p* < 0.01), along with significant reductions in E2 and P4 levels (*p* < 0.05). However, overexpression of *FOSL2* significantly increased *FOSL2* expression (*p* < 0.0001) and *Trib1* expression remained unchanged (*p* > 0.05). In addition, *Star* and *Cyp11a1* expression levels were significantly elevated (*p* < 0.0001), accompanied by increased E2 and P4 secretion (*p* < 0.05). Compared with the siTrib1 group, co-transfecting *FOSL2* and siTrib1 group restored the expression levels of *Star* and *Cyp11a1* (*p* < 0.0001) and rescued the reductions in E2 and P4 secretion caused by *Trib1* knockdown (*p* < 0.05) (Figure 6G–L). These results demonstrate that overexpression of *FOSL2* compensates for the downregulation of *Star* and *Cyp11a1* expression caused by *Trib1* knockdown and restores the reduced E2 and P4 levels.

## 4. Discussion

*Trib1*, a member of the Tribbles family, was initially identified as a regulator of gastrulation cell proliferation in Drosophila embryos [16]. The protein sequence and structure of *Trib1* are highly conserved across species, suggesting functional conservation [13,17]. Based on the expression patterns of *Trib1* in the ovaries during the follicular phase across different ages, we found that, to some extent, the trend in *Trib1* expression levels in the ovary is positively correlated with reproductive capacity, and the TRIB1 protein was predominantly expressed in ovarian GCs. This suggests that *Trib1* plays a crucial role in GCs and is involved in regulating ovarian reproductive performance.

GCs are the principal somatic cell type within the follicle, and their steroid hormone synthesis capacity is particularly important for normal mammalian reproduction. GCs are mainly responsible for the conversion of androgens to estrogen and the synthesis of P4 [18]. Steroid hormone biosynthesis begins with the transport of intracellular cholesterol across the mitochondrial membrane by the Steroidogenic acute regulatory protein (*Star*). *Cyp11a1* is responsible for cutting off the cholesterol side chain and converting it to pregnenolone, and *Cyp11a1* is the only enzyme in this process, which determines the steroidogenic ability of GCs [19,20]. Then, *Hsd3b* in the endoplasmic reticulum converts pregnenolone to P4 [21]. *Cyp17a1* can catalyze pregnenolone and progesterone to form androstenedione [22], which is then converted by *Cyp19a1* to estrogen [23]. *Cyp19a1* is primarily expressed in the GCs of mature follicles [24]. Inhibition of *Cyp19a1* expression in GCs reduces estrogen secretion, which can lead to premature follicular atresia [1]. *Trib1* functions as a molecular scaffold that modulates MAPK and AKT signaling to promote tumor cell proliferation [25]. In adipocytes, *Trib1* directly regulates lipid metabolism. Emerging evidence shows that *Trib1* alters mitochondrial respiratory chain complex enzyme activities, disturbing mitochondrial fusion/fission balance and leading to mitochondrial damage and lipid metabolic dysfunction [26]. In this study, *Trib1* knockdown in ovarian GCs significantly reduced the expression of steroidogenesis-related genes, including *Star*, *Cyp11a1*, *Hsd3b1*, *Hsd17b7*, and *Cyp19a1*, accompanied by markedly decreased E2 and P4 levels, indicating that *Trib1* is critical for steroid hormone synthesis and secretion in ovarian GCs.

To investigate the biological function of the *Trib1* gene in vivo and its effects on female fertility, this study generated a *Trib1* KO mouse model. Comprehensive evaluation of reproductive capacity, ovarian endocrine function, and reproductive potential provided critical insights into the role of *Trib1* in ovarian function. Litter size during specific reproductive cycles serves as a key indicator of female fertility [27], while the number of mature follicles reflects ovarian reserve and reproductive performance [28]. During follicular development, most follicles undergo atresia at the antral stage, with only a small fraction advancing to ovulation [29]. E2, synthesized primarily by GCs, is closely associated with follicular development and number [30] and regulates the development and selection of dominant follicles prior to ovulation, with insufficient E2 levels potentially leading to premature follicular atresia [31,32]. This study found that *Trib1*−/− female mice exhibited slow growth, reduced body weight, and complete infertility. Furthermore, interbreeding of *Trib1*+/− mice resulted in offspring genotype distributions that significantly deviated from Mendelian inheritance, with *Trib1*−/− offspring far below the expected ratio. Ovarian dissection revealed that *Trib1*−/− mice had reduced ovarian volume and ovarian index, fewer mature follicles, and an increased number of atretic follicles. Serum levels of E2 and P4 were significantly reduced, consistent with observations from *Trib1* knockdown in GCs. These results indicate that *Trib1* deletion severely impairs female fertility by suppressing GC steroid hormone synthesis, disrupts follicular development and maturation, and reduces ovarian reserve. This highlights the critical role of *Trib1* in follicular development and steroidogenesis in female mice. Because this study used a whole-body *Trib1* knockout mouse model, we cannot exclude the possibility that *Trib1* deletion in other tissues affects female reproductive performance. Nevertheless, the results indicate that *Trib1* plays a crucial role in female fertility and is important for steroid hormone synthesis in ovarian granulosa cells. To further delineate the unbiased contribution of *Trib1* impact on female reproduction in vivo, generating a conditional *Trib1* knockout model would be the most appropriate approach and we will pursue this in our future work.

*Trib1* is a member of a family of serine/threonine pseudokinase proteins, and all members share a unique central kinase-like domain with a variable N-terminal and a (largely) conserved C-terminal on either side [33]. The “PEST” region in the N-terminus of the TRIB proteins family has been identified as a key feature contributing to proteins’ instability [34]. *Trib1* is a highly unstable protein, a property crucial for its regulatory functions, allowing for rapid changes during cellular responses [35,36]. This instability posed significant challenges for detecting and quantifying TRIB1 protein levels in our experiments. Despite multiple attempts, *Trib1* was difficult to detect and quantify; therefore, in vitro detection results are not included in this study. According to the literature, *Trib1* can form complexes with various transcription factors, modulating transcriptional regulation [37], and also modulates signaling pathways by activating or inhibiting specific kinases, thereby influencing phosphorylation, nuclear translocation, and expression of downstream transcription factors [38]. Notably, STRING database predictions indicate an interaction between *Trib1* and the transcription factor *FOSL2*. *Trib1* and *FOSL2* exhibit highly consistent expression patterns in ovarian GCs, and knockdown of *Trib1* in GCs significantly reduces *FOSL2* expression. Furthermore, their FPKM values from ovarian RNA-Seq data show a significant positive correlation, and *FOSL2* expression is markedly reduced in the ovaries of *Trib1* KO mice. These findings strongly suggest a close functional relationship between *Trib1* and *FOSL2*.

*FOSL2* is a member of the Fos family and a part of the activating protein 1 (AP-1) transcription complex. The AP-1 family is one of the most widely studied transcription factors families. AP-1 regulates gene expression by binding to specific DNA sequences, thereby modulating transcription [39]. Studies have shown that *FOSL2* KO mice exhibit no differences at birth compared to wild-type mice, but *FOSL2* KO mouse pups display severe growth defects and die within the first week [40]. To further investigate the role of *FOSL2* in ovarian GCs, Zhang et al. generated a GC-specific *FOSL2* deletion mice model (Cyp19a1-Cre; FOSL2 Flox/Flox, *FOSL2* CKO mice), and *FOSL2* CKO mice exhibited significantly reduced reproductive capacity, with a notable decrease in both the quantity and quality of ovulation. Additionally, these mice showed a marked reduction in sensitivity to gonadotropins, highlighting the critical role of *FOSL2* in follicular development and ovulation processes in mice [41]. Our results predicted strong *FOSL2* motif binding sites within the promoter regions of *Star* and *Cyp11a1*. In *Trib1* knockdown GCs, *FOSL2* overexpression significantly increased *Star* and *Cyp11a1* expression, restoring steroid hormone synthesis. Co-transfection of the pcDNA3.1-FOSL2 plasmid in Trib1-knockdown ovarian granulosa cells partially restored their steroidogenic capacity, with changes in *CYP11A1*, E2, and P4 aligning with expectations, though *STAR* expression showed some deviations. It is widely recognized that the synthesis of steroid hormones in these cells involves a complex and finely tuned regulatory mechanism, influenced by numerous factors. Therefore, when both siTrib1 and pcDNA3.1-FOSL2 are co-transfected into ovarian GCs, the effects of *FOSL2* overexpression may overshadow the impact of *Trib1* knockdown, indicating a quantitative relationship in their regulatory interaction. This insight offers new directions for our future research, enabling us to further explore the molecular mechanisms of *Trib1* and *FOSL2* in ovarian GCs.

## 5. Conclusions

In this study, TRIB1 protein is predominantly expressed in GCs. Knockdown of *Trib1* in GCs significantly downregulates genes involved in steroidogenesis and markedly reduces E2 and P4 secretion. Female *Trib1* KO mice exhibit developmental delay and ovarian dysfunction, along with a decrease in the number of antral follicles and a marked increase in atretic follicles, ultimately resulting in complete infertility. DEGs of *Trib1* KO ovaries were enriched in steroid biosynthesis pathways, associated with significant downregulation of *FOSL2.* In vitro, overexpression of *FOSL2* rescues their steroidogenic capacity mediated by knockdown of *Trib1*. Collectively, these data indicate that the *Trib1* gene regulates steroidogenesis in GCs and is essential for fertility in female mice, which provides profound insights into female reproductive endocrinology and potential therapeutic targets.

## Figures and Tables

**Table 1:** Primer sequences for genotyping of *Trib1* KO mice.

Name	Sequence (5′ → 3′)	Product Length (bp)
Primers 1	F1: GCTTGGGTTTGGCAGAGCAGATAAG	Targeted allele: 530 bp; Wildtype allele: 3519 bp
	R1: GTGCTAACTTCGGTATGTCCTCAGC	
Primers 2	F1: GCTTGGGTTTGGCAGAGCAGATAAG	Homozygotes: 530 bp; Heterozygotes: 530 bp/600 bp; Wildtype allele: 600 bp
	R2: AGACCGGCCATCCTGTATCTAGTTG	

**Table 2:** RT-qPCR Primer Information for Steroid Hormone-Related Marker Genes.

Gene Name	Primer Sequences	NCBI Reference Sequence	Product Length/bp
*Trib1*	F: TCCCATAGCAACATCACTGGC	NM_144549.4	110
	R: TTTCGGCTCCGCACATAGGA		
*Star*	F: CTTGGCTGCTCAGTATTGAC	NM_011485.5	153
	R: TGGTGGACAGTCCTTAACAC		
*Cyp11a*	F: CGATACTCTTCTCATGCGAG	NM_001346787.1	126
	R: CTTTCTTCCAGGCATCTGAAC		
*Hsd3b1*	F: AGCTCTGGACAAAGTATTCCGA	NM_001304800.1	234
	R: GCCTCCAATAGGTTCTGGGT		
*Hsd17b1*	F: ACTTGGCTGTTCGCCTAGC	NM_010475.2	117
	R: GAGGGCATCCTTGAGTCCTG		
*Hsd17b7*	F: CCTCTCGCAATGCAAAGAAGG	NM_001420236.1	98
	R: GAGGTCGGTAGCATATTTGGAAG		
*Hsd17b12*	F: GGCTTCCTGTACTGGGTGG	NM_019657.4	196
	R: CACGTTTTGCTAACTCTTCTGC		
*Cyp17a1*	F: GGCCATGGCAGGAAACTACT	NM_007809.3	159
	R: GCCCAAGTCAAAGACACCTAAT		
	R: GTACCCAGGCGAAGAGAATAGA		
*Cyp19a1*	F: GACACATCATGCTGGACACC	NM_001348171.1	179
	R: CAAGTCCTTGACGGATCGTT		
*Gapdh*	F: GAGAGTGTTTCCTCGTCCCGTA	NM_001289726.2	143
	R: TGAGGTCAATGAAGGGGTCG		

**Table 3:** siRNA sequences targeting the mouse *Trib1*.

Name	Sequence (5′ → 3′)
siRNA	F: GAACUUCAGACCAGAUUGUTT
	R: ACAAUCUGGUCUGAAGUUCTT
Negative control (NC)	F: UUCUCCGAACGUGUCACGUTT
	R: ACGUGACACGUUCGGAGAATT

**Table 4:** RT-qPCR Primer Information for DEGs from RNA-Seq.

Gene Name	Primer Sequences	NCBI Reference Sequence	Product Length/bp
Aqp2	F: AACTCCGGTCCATAGCGTTC	NM_009699.3	263
	R: CACGTAGAAGGCAGCTCGAA		
Itgal	F: AGCTACAACCTGGACACACG	XM_006507393.5	187
	R: AACCATGTAGGCTGACTGGC		
Epha4	F: GGAGAGCTTGGGTGGATAGC	NM_007936.3	150
	R: GGTGATCCAGTCAGTTCGCA		
Enpp2	F: TCTAGCATCCCAGAGCACCT	NM_001411655.1	265
	R: CCCCATTCCTTTCTGACGCA		
Itih2	F: TGCCTCAGAGTGTCGTGTTC	NM_010582.4	120
	R: CTCGGCCCACAGTTTTCTCT		
Bpifb4	F: GGTGTCCCGTACAACGACTT	NM_001034875.4	196
	R: GACGGTAGTCTTCATGCCGT		
Nrcam	F: GGTGCAGGCAAAGGCAAAGA	NM_176930.4	169
	R: TTGCTGGTCCTGTCTCAAACAC		
Cemip	F: GGCCATGGCAGGAAACTACT	NM_030728.4	175
	R: CAAAAGGGCAAAGGGCACTC		
Abcb1b	F: CCTGCTGTTGGCGTATTTGG	NM_011075.2	122
	R: AACACCAGCATCAAGAGGGG		
Lipg	F: GGCGGGAAGTATCACAACCT	NM_010720.3	125
	R: CTGGGTCCTTAGAAGTGCGG		
Ptgfr	F: ATAATGTGCGTCTCCTGCGT	NM_008966.3	132
	R: CTGATTCCACGTTGCCATGC		
Sfrp4	F: GTACGCACCCATCTGTACCC	NM_016687.4	155
	R: CATAGACCGGCAGCTCATCG		
Gapdh	F: GAGAGTGTTTCCTCGTCCCGTA	NM_001289726.2	143
	R: TGAGGTCAATGAAGGGGTCG		

**Table 5:** Genotype distribution among live offspring of *Trib1* intercross.

Parental *Trib1* Genotype	Total Offsprings	Genotype *p* = 0.000017		
		+/+ N (%)	+/− N (%)	−/− N (%)
Expected	/	(25)	(50)	(25)
Observed	176	62 (35)	97 (55)	17 (10)

## Data Availability

The raw sequence data reported in this paper have been deposited in the Genome Se-quence Archive (Genomics, Proteomics & Bioinformatics 2021) in National Genomics Data Center (Nucleic Acids Res 2022), China National Center for Bioinformation/Beijing Institute of Genomics, Chinese Academy of Sciences (GSA: PRJCA042504) that are publicly accessible at https://ngdc.cncb.ac.cn/gsa. URL (accessed on 2 July 2025).

## Abbreviations

The following abbreviations are used in this manuscript:

GCs	Granulosa cells
TCs	Theca cells
CL	Corpus luteum
E2	Estradiol
P4	Progesterone
RNA-Seq	RNA sequencing
DEGs	Differentially expressed genes
